# Supplementary material for: Numerosity estimation of virtual humans as a digital-robotic marker for hallucinations in Parkinson’s disease
Source: Nat Commun. 2024 Mar 12;15:1905. doi: 10.1038/s41467-024-45912-w (PMC10933269; doi:10.1038/s41467-024-45912-w)
Supplement: Supplementary file 1 — Supplementary Information [file 41467_2024_45912_MOESM1_ESM.pdf]

Supplementary Information

Numerosity estimation of virtual humans as a digital-robotic marker for hallucinations in Parkinson's disease

Authors

Louis Albert[1], Jevita Potheegadoo[1], Bruno Herbelin[1], Fosco Bernasconi[1], Olaf Blanke[1,2]

Affiliations

[1] Laboratory of Cognitive Neuroscience, Neuro-X Institute, Faculty of Life Sciences, Swiss Federal Institute of Technology (EPFL), Geneva, Switzerland

[2] Department of Clinical Neurosciences, Faculty of Medicine, University of Geneva, Geneva, Switzerland

Corresponding Author

Olaf Blanke

Bertarelli Chair in Cognitive Neuroprosthetics

Neuro-X Institute

School of Life Sciences

Campus Biotech

Swiss Federal Institute of Technology

Ecole Polytechnique Fédérale de Lausanne (EPFL)

CH – 1012 Geneva

E-mail: olaf.blanke@epfl.ch

Tel: +41 (0)21 693 69 21

Keywords

Neurodegeneration, Virtual Reality, Robotics, Web-based assessment, Psychosis

Supplementary Note 1: Human numerosity estimation subitizing range calculation (online pilot study)

An online web-based experiment was developed to determine the subitizing range of human numerosity stimuli, and thus select the range of stimuli to display in study 1. We aimed at selecting a range of stimuli in the early range of numerosity estimation process (just above the subitizing range), where numerosity estimation errors start to occur.

Thus, when the presence hallucination is induced, this felt presence (which is not visual) would interact with the uncertainty of numerosity estimation of virtual human agents and push the estimation system of human agents toward the upper number. This would not be the case when boxes are shown. Participants first filled in some socio-demographic information (gender and age), followed by the human numerosity estimation task. This experiment was available in French and English.

Study population

Twenty-height healthy participants (16 women, 12 men; age ranging from 21 to 42 years, mean ± SD age = 29.5 ± 6.13 years) took part in the online web-based experiment.

Numerosity stimuli

Visual stimuli were generated on Unity 3D (version 2019.3.13f1). The virtual environment was modeled in 3DS max and consisted of a realistic representation of our experimental room. Stimuli consisted of a 3D scene of our virtual experiment room with virtual human agents inside (examples in Supplementary Figure 2a, Supplementary Figure 2c, Supplementary Figure 2e, Supplementary Figure 2g). Virtual human agents were placed in the virtual scene in front of the camera viewpoint, facing the viewpoint in a range from – 90° to 90°, and in a way that they do not overlap completely from the viewpoint. Virtual human agents were placed between 1.75m and 5.25m in depth from viewpoint, and between -1.5m and 1.5m from right to left. The

array of virtual human agents occupied a maximum virtual camera visual angle of 60° horizontally. This ensures that virtual human agents and control objects were located within the participants' peripheral field of view, which is the "vision produced by light falling on areas of the retina outside the macula" [1], and that they were displayed inside and close to the limit of 30° retinal eccentricity, which is the limit from which the visual acuity decreases more strongly [2]. The number of virtual human agents ranged from 1 to 24.

Procedure

Participants first filled in some socio-demographic information (gender and age). Then, before starting the human numerosity estimation task, participants performed a screen calibration procedure, which consisted in measuring and reporting the length of a line displayed on their screen. This measure allowed to scale stimuli, so they were displayed the same physical size on each participant screen, independent of monitor screen and positions. Participants were instructed to stay fifty centimeters away from their screen. The human numerosity estimation task contained 120 trials, 5 trials per numerosity (ranging from 1 to 24), randomized order between participants. Before each trial, participants were asked to fix their gaze on a fixation cross, drawn from a uniform distribution between 700 and 1500 msec (step of 200 msec). The total duration of the experiment was approximately 10 minutes.

Data analysis

The error rate (ER), mean response time (RT), mean response, and variation coefficient (VC; standard deviation of response divided by mean response) were calculated for each numerosity and each participant. A stable VC across numerosity reflects scalar variability and Weber's law, which is a signature of estimation processes.

The proxy for the subitizing range was calculated using the ISR algorithm [3]. The data for RT (numerosity 1 to 7 [4]) were used to fit a sigmoid function with unknown parameters using the Levenberg-Marquardt non-linear fitting algorithm [5]. The proxy for the subitizing range was

taken as the intersection point between the tangent at the inflection point of the sigmoid curve and a line with a slope of zero and an intercept where the sigmoid curve crosses the y-axis at x = 0 (i.e., the subitizing line). This represents the point at which the slope begins to appreciably change, thus representing an accurate estimate of the upper bound of the subitizing range [3].

Linear mixed effects models (packages lme4 [6] and lmerTest [7] in R [8] with numerosity as a fixed effect and participant as a random effect were then used to examine the limit when participants' patterns of performance obeyed Weber's law by showing scalar variability (i.e., constant coefficients of variation), to confirm when estimation mechanisms were active.

Preprocessing

For each participant, trials in which response time were superior to the mean plus three standard deviations were excluded, resulting in the exclusion of 1.55% of the trials across conditions and participants (52 trials over 3360). Trials in which participants answer contains three digits were excluded, as well as trials in which the participants answer contains two digits when the presented numerosity was in the subitizing range, resulting in the exclusion of 0.12% of the trials (4 trials over 3300).

Results

Descriptive statistics

To demonstrate the expected pattern of numerosity task, we plotted RT, ER and VC as a function of numerosity (Supplementary Figure 15). Errors were very rare for numerosity 1 to 4 and then the error rate steeply increased with numerosity. RT gradually increased from numerosity 1 to 8 with a slope that became steeper with numerosity between numerosity 1 to 5, and with a slope that then became less steep with numerosity between numerosity 5 to 8. After numerosity 8, RT reaches a kind of plateau. This sigmoidal RT curve behavior is typically observed in numerosity experiment [9].

ISR calculation

We calculated ISR according to RT for correct responses only. Three participants had R^2 value of 0.7 or less, which were considered unreliable [3] and they were excluded from further analysis. Mean R^2 value for the rest of the participants was 0.88, with a standard deviation of 0.08. This analysis yielded a mean subitizing range of 3.31 (SD = 1.19).

Scalar variability

We ran a series of linear mixed effects models on coefficient of variation data with numerosity as a fixed effect and participant as a random effect. With data considering numerosity 1 to 24 (respectively 2 to 24, 3 to 24 and 4 to 24), the difference was statistically different ($F(1,642)=155.97$, $p<0.001$), respectively $F(1,614)=96.89$, $p<0.001$), $F(1,5862)=47.27$, $p<0.001$) and $F(1,558)=16.18$, $p<0.001$)). With data considering numerosity 5 and above to 24, the difference was not significant anymore.

Lower bound of the estimation range

The limit when participants' patterns of performance obeyed Weber's law by showing scalar variability (i.e., constant coefficients of variation) was numerosity 5. This result, coupled with the proxy result of subitizing range calculated with the ISR gave us the lower bound of the estimation range at numerosity 5.

Apparatus and material

This online experiment was developed in house using javascript (on client side: javascript, html, css; on server side: https server in node.js, nginx as a reverse proxy, running in two docker containers) and was hosted on an EPFL Server in a dedicated Virtual Machine (1xvCPU, 1GB RAM, 40GB HDD) in Demilitarized Zone. Data was saved and stored on EPFL’s servers.

Ethics

This online study, as being irreversibly anonymous, was considered as falling outside of the scope of the Swiss legislation regulating research on human subjects, so that the need for local ethics committee approval was waived.

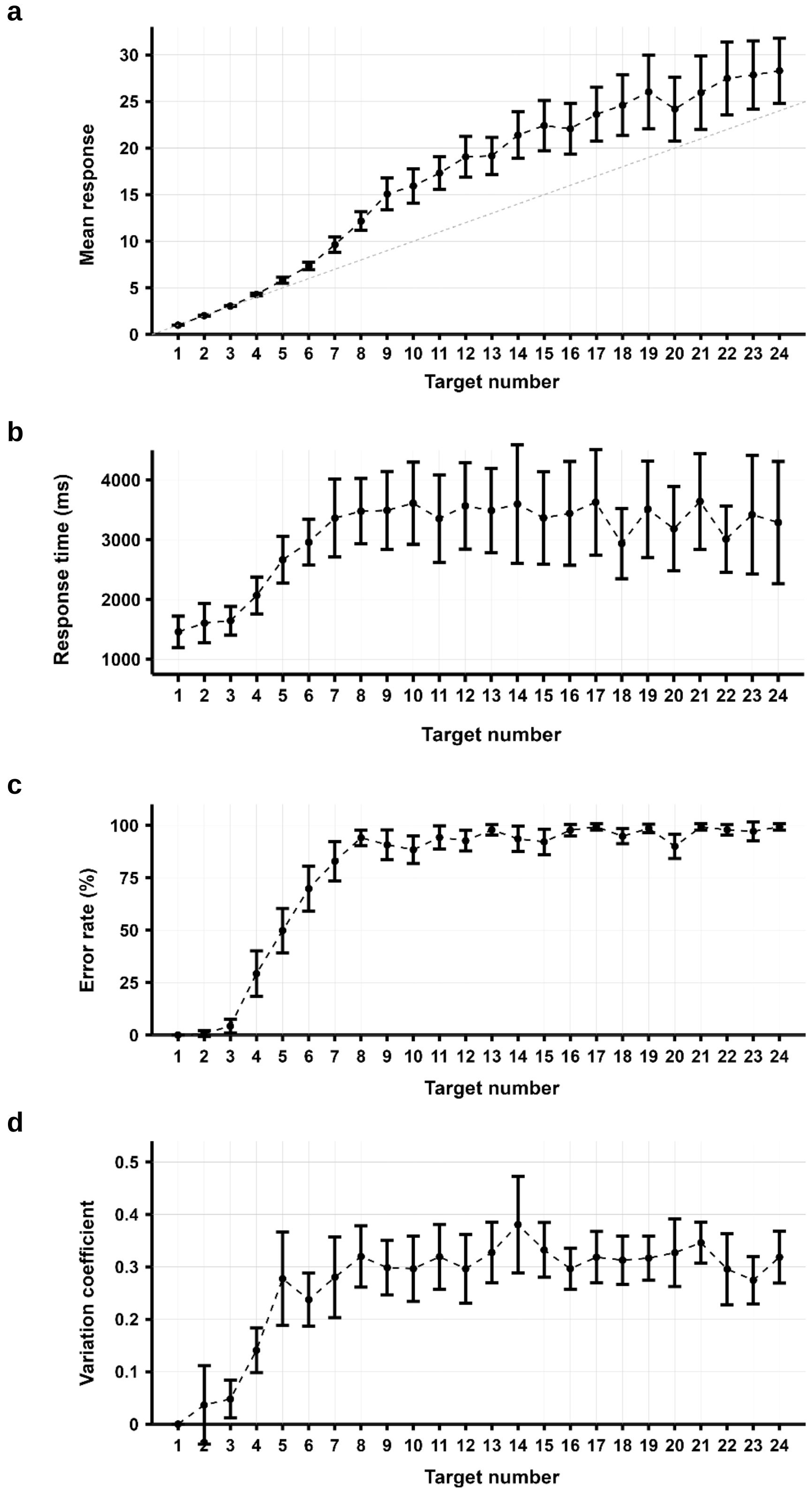

Supplementary Figure 15. Online pilot study results. Subitizing range calculation experiment – Results. Mean response (a), response time (b), error rate (c) and variation

coefficient (d) as a function of presented numerosity. Error bars represent 95 CI. n = 28 healthy participants. Source data are provided as a Source Data file.

Supplementary Note 2: Mediation analysis – human numerosity estimation task (study 1)

Step 1:

Supplementary Table 12. Effect of the robotic sensorimotor stimulation on human numerosity estimation (Human Numerosity Estimation task).

In step one of the mediation model, the effect of the robotic sensorimotor stimulation on human numerosity estimation, ignoring the mediator (robot-induced presence hallucination question rating), was significant (F(1,27)=26.05; p = 2.3e-5).

	Sum Sq	Mean Sq	NumDF	DenDF	F value	Pr(>F)
Robotic Sensorimotor Stimulation	0.31	0.31	1	27	26.05	2.3e-5

	NEH		
**Predictors**	**Estimates**	**CI**	**p**
**(Intercept)**	6.82	6.62 – 7.20	<2e-16
Robotic Sensorimotor Stimulation	0.15	0.09 – 0.21	2.3e-5
**Random effects**			
**σ2**	0.01		
τ00 S_ID	0.27		
ICC	0.96		
N S_ID	28		
Observations	56		
Marginal R2 / Conditional R2	0.019/0.959		

Step 2:

Supplementary Table 13. Effect of the robotic sensorimotor stimulation on robot-induced presence hallucination question rating (Human Numerosity Estimation task).

Step two showed that the effect of the robotic sensorimotor stimulation on the mediator (robot-induced presence hallucination question rating), was significant (F(1,27)=10.44;p=0.003).

	Sum Sq	Mean Sq	NumDF	DenDF	F value	Pr(>F)
Robotic Sensorimotor Stimulation	8.64	8.64	1	27	10.44	0.003

	robot-induced PH question rating		
**Predictors**	**Estimates**	**CI**	**p**
**(Intercept)**	1.5	0.75 – 2.25	0.0003
Robotic Sensorimotor Stimulation	0.79	0.30 – 1.27	0.002

Random effects	
σ2	0.83
τ00 S_ID	3.07
ICC	0.79
N S_ID	28
Observations	56
Marginal R2 / Conditional R2	0.039/0.796

Step 3:

Supplementary Table 14. Effect of the robot-induced presence hallucination question rating on human numerosity estimation, controlling for robotic sensorimotor stimulation (Human Numerosity Estimation task).

Step three of the mediation process showed that the effect of the mediator (robot-induced presence hallucination question rating), controlling for robotic sensorimotor stimulation, was significant ($F(1,35)=6.53$; $p=0.02$). The effect of the robotic sensorimotor stimulation was also still significant ($F(1,28)=11.80$; $p=0.002$).

	Sum Sq	Mean Sq	NumDF	DenDF	F value	Pr(>F)
Robotic Sensorimotor Stimulation	0.12	0.12	1	28.13	11.80	0.002
robot-induced PH question rating	0.07	0.07	1	34.89	6.53	0.015

		NEH	
**Predictors**	**Estimates**	**CI**	**p**
**(Intercept)**	6.75	6.54 – 6.95	<2e-16
Robotic Sensorimotor Stimulation	0.11	0.04 – 0.17	0.001
robot-induced PH question rating	0.05	0.01 – 0.09	0.014

Random effects	
σ2	0.01
τ00 S_ID	0.25
ICC	0.96
N S_ID	28
Observations	56
Marginal R2 / Conditional R2	0.056/0.962

Step 4:

Supplementary Table 15. Causal mediation analysis (Human Numerosity Estimation task).

ACME stands for average causal mediation effects (indirect effect of the robotic sensorimotor stimulation on human numerosity estimation), ADE stands for average direct effects (direct effect of the robotic sensorimotor stimulation on human numerosity estimation), Total Effect stands for the total effect (direct plus indirect effect of the robotic sensorimotor stimulation onto human numerosity estimation), Prop. Mediated describes the proportion of the effect of the robotic sensorimotor stimulation on human numerosity estimation that goes through the robot-induced presence hallucination question rating.

	**Estimates**	**95% CI lower**	**95% CI upper**	**p-value**
**ACME**	0.04	0.006	0.08	0.01
ADE Stimulation	0.11	0.05	0.17	<2e-16
Total Effect	0.15	0.09	0.21	<2e-16
Pop. mediated	0.26	0.04	0.56	0.01

Supplementary Note 3: Mediation analysis object numerosity estimation task (study 1)

Step 1:

Supplementary Table 16. Effect of the robotic sensorimotor stimulation on object numerosity estimation (Object Numerosity Estimation task).

In step one of the mediation model, the effect of the robotic sensorimotor stimulation on object numerosity estimation, ignoring the mediator (robot-induced presence hallucination question rating), was not significant ($F(1,27)=3.71$; $p=0.06$).

	Sum Sq	Mean Sq	NumDF	DenDF	F value	Pr(>F)
Robotic Sensorimotor Stimulation	0.13	0.13	1	27	3.71	0.065

	NEO		
**Predictors**	**Estimates**	**CI**	**p**
**(Intercept)**	6.98	6.78 – 7.18	<2e-16
Robotic Sensorimotor Stimulation	-0.09	-0.19 – 0.00	0.06
**Random effects**			
σ2	0.03		
τ00 S_ID	0.25		
ICC	0.88		
N S_ID	28		
Observations	56		
Marginal R2 / Conditional R2	0.008/0.883		

Step 2:

Supplementary Table 17. Effect of the robotic sensorimotor stimulation on robot-induced presence hallucination question rating (Object Numerosity Estimation task).

Step two showed that the effect of the robotic sensorimotor stimulation on the mediator (robot-induced presence hallucination question rating), was significant ($F(1,27)=10.44$;$p=0.003$).

	Sum Sq	Mean Sq	NumDF	DenDF	F value	Pr(>F)
Robotic Sensorimotor Stimulation	8.64	8.64	1	27	10.44	0.003

	robot-induced PH question rating		
**Predictors**	**Estimates**	**CI**	**p**
**(Intercept)**	1.5	0.75 – 2.25	0.0003
Robotic Sensorimotor Stimulation	0.79	0.30 – 1.27	0.002

Random effects	
σ2	0.83
τ00 S_ID	3.07
ICC	0.79
N S_ID	28
Observations	56
Marginal R2 / Conditional R2	0.039/0.796

Step 3:

Supplementary Table 18. Effect of the robot-induced presence hallucination question rating on object numerosity estimation, controlling for robotic sensorimotor stimulation (Object Numerosity Estimation task).

Step three of the mediation process showed that the effect of the mediator (robot-induced presence hallucination question rating), controlling for robotic sensorimotor stimulation, was not significant ($F(1,47)=0.008$; $p=0.93$). The effect of the robotic sensorimotor stimulation was also not significant ($F(1,28)=2.78$; $p=0.11$).

	Sum Sq	Mean Sq	NumDF	DenDF	F value	Pr(>F)
Robotic Sensorimotor Stimulation	0.10	0.10	1	27.63	2.78	0.11
robot-induced PH question rating	0.0003	0.0003	1	47.24	0.008	0.93

		NEO	
**Predictors**	**Estimates**	**CI**	**p**
**(Intercept)**	6.98	6.76 – 7.21	<2e-16
Robotic Sensorimotor Stimulation	-0.309	-0.20 – 0.02	0.11
robot-induced PH question rating	-0.00	-0.07 – 0.06	0.93

Random effects	
σ2	0.03
τ00 S_ID	0.26
ICC	0.88
N S_ID	28
Observations	56
Marginal R2 / Conditional R2	0.008/0.883

Step 4:

Supplementary Table 19. Causal mediation analysis (Object Numerosity Estimation task).

ACME stands for average causal mediation effects (indirect effect of the robotic sensorimotor stimulation on object numerosity estimation), ADE stands for average direct effects (direct effect of the robotic sensorimotor stimulation on object numerosity estimation), Total Effect stands for the total effect (direct plus indirect effect of the robotic sensorimotor stimulation onto object numerosity estimation), Prop. Mediated describes the proportion of the effect of the robotic sensorimotor stimulation on object numerosity estimation that goes through the robot-induced presence hallucination question rating.

	Estimates	95% CI lower	95% CI upper	p-value
**ACME**	-0.002	-0.06	0.05	0.92
ADE Stimulation	-0.09	-0.20	0.02	0.10
Total Effect	-0.09	-0.19	0.00	0.06
Pop. mediated	0.02	-1.19	1.18	0.93

Supplementary Note 4: Prevalence of hallucinations (study 2)

Over our 170 patients, 63% (108/170) reported having experienced hallucinatory phenomena (passage hallucinations, presence hallucinations, visual illusions or complex visual hallucinations). Although the prevalence of hallucinatory symptoms in PD varies remarkably through studies, evidence from previous works suggest that minor hallucinations may occur in up to 72% of patients[10–12], with presence hallucinations and passage hallucinations being the most frequent minor hallucinations [11,13,14]. Our results corroborate this evidence with 63% (107/170) of our PD patients having experienced minor hallucinations. The most frequent type of minor hallucinations was passage hallucinations in 53% of patients (90/170), followed by presence hallucinations in 41% of patients (69/170). The frequencies and occurrences of the different forms of hallucinations are shown in Supplementary Figure 13 and reported in Supplementary Table 8.

Supplementary Note 5: Effects of clinical variables on presence hallucination in PD and numerosity estimation (study 2)

To ensure that the observed differences between human numerosity estimation and object numerosity estimation could not be explained by a difference in demographic and clinical variables, we built a linear mixed effects model with online numerosity estimation magnitude as the outcome variable, PD group (PD-nH and PD-PH), presented numerosity and type of stimulus (virtual human agents or control objects) as fixed effect, age, gender, disease duration and first affected side as covariate fixed effects, and a random intercept for each subject. Our results show no influence of these covariates on numerosity estimation (age ($F(1, 110)=0.66$; $p=0.42$); gender ($F(1, 110)=2.30$; $p=0.13$); disease duration ($F(1, 110)=2.79$; $p=0.10$); first affected side ($F(1, 110)=0.95$; $p=0.42$)) (Supplementary Table 10), strengthening our argumentation that the selective online human numerosity estimation effect in PD patients is directly linked to experiencing presence hallucinations.

Supplementary Note 6: Online human numerosity estimation and online object numerosity estimation response time differences (study 2)

Additional analysis ensured that the observed differences between online human numerosity estimation and online object numerosity estimation are not explained by differences in task difficulty between groups. Thus, a linear mixed effects model with response time as the outcome variable, PD group (PD-nH and PD-PH), presented numerosity and type of stimulus (humans or control objects) as fixed effect, and a random intercept for each subject (Supplementary Table 11) showed no differences in response times between the two PD groups (i.e., PD group ($F(1,116) = 0.58$; $p=0.45$); no main effect nor any interaction), suggesting each task (human and object numerosity estimation tasks) difficulty is equal between our two PD groups (PD-nH and PD-PH). However, our results indicate a main effect of type of stimuli ($F(1,9084)=78.67$; $p = 8.7e-19$), with participants being faster on the online object numerosity estimation task compared to online human numerosity estimation task (Supplementary Table 11). This main effect was not expected, and suggest that, for our PD patients' sample, the object numerosity estimation task is easier compared to the human numerosity estimation task. This is supported by the fact PD patients gives higher human numerosity estimation than object numerosity estimation (main effect of Type of Stimuli ($F(1,9085)=1188$; $p = 7.9e-245$) in the previous model with online numerosity estimation magnitude as the outcome variable, see Supplementary Table 9). Although unexpected, we argue that this difference alone cannot explain the overestimation observed in PD-PH versus PD-nH and in online human numerosity estimation task versus online object numerosity estimation task, which is more prone to be intrinsically linked to the experience of spontaneous presence hallucinations. Indeed, our observations follow our findings from study 1, with PD-PH overestimating human numerosity estimation as compared to PD-nH. As in study 1, we observed no differences in object numerosity estimation between groups. Furthermore, our results indicating no difference in response time between our two PD groups, this suggests that despite the fact the object numerosity estimation task is easier compared to the human

numerosity estimation task in our patient's sample, each task difficulty is equal between our two PD groups (PD-nH and PD-PH). Taken together, this suggests that the overestimation observed in PD-PH versus PD-nH and in online human numerosity estimation task versus online object numerosity estimation task cannot be explained by a difference in task difficulty but is intrinsically linked to the experience of spontaneous presence hallucinations.

Supplementary Note 7: online study dropout rate (study 2)

13 PD patients (13/183 = 7%) stopped the experiment during the numerosity estimation task. All drops were made during the first half of the numerosity estimation task (trials where drop happened: 3; 10; 14; 17; 20; 20; 22; 22; 25; 32; 38; 40; 40 – median = 22).

Participants could stop the experiment whenever they wanted, and they could indicate a reason for the dropout by ticking a box.

Drop reason	count
The experiment takes too much time	5
The task is too complicated	0
The experiment is boring	4
I am too tired to continue	1
I am not satisfied with my answers	0
Other (not indicated)	3

Supplementary Table 20. Online study dropout rate.

Supplementary Note 8: Symptoms and effects induced (study 1)

At the end of the experiment, once the VR headset was removed, participants were assessed with a questionnaire about symptoms and effects induced by the experiment and virtual reality. This questionnaire contained 5 items ("Did you feel nauseous?" ; "Did you feel headaches?" ; "Did you feel dizzy?" ; "Did you feed tired?" ; "Have you experienced any loss of balance?") rated on a 7-item Likert scale. 1 corresponds to "Extremely intense feeling", 2 to "Very intense feeling", 3 to "Intense feeling", 4 to "Moderate feeling", 5 to "Weak feeling", 6 to "Very weak feeling", and 7 to "Absent". In addition to these 5 items, participants were presented with one text zone of free report on "Other symptoms / Notes / Suggestions". The results are shown in Supplementary Table 21 and Supplementary Figure 16.

Question	Mean	*SD*	95% CI lower	95% CI upper	Corresponding feeling
**Did you feel nauseous?**	6.79	0.5	6.59	6.98	Absent
**Did you feel headaches?**	6.25	1.11	5.82	6.68	Very weak feeling
**Did you feel dizzy?**	5.93	1.15	5.48	6.38	Very weak feeling
**Did you feed tired?**	3.93	1.09	3.51	4.35	Moderate feeling
**Have you experienced any loss of balance?**	6.57	0.63	6.33	6.82	Absent

Supplementary Table 21. Summary of participants responses to symptoms and effects induced questionnaire reported in study 1.

In addition, 6 of the 28 total participants tested made a remark in the "Other symptoms / Notes / Suggestions":

- "For headaches, lower now that IU have removed the headset"
- "Pain in my neck"
- "Back ache, due to the sitting down position"
- "Arm ache, back ache (due to the moving of the arm)"
- "I had pain in the neck during the experiment due to the weight of headphones"
- "Neck stiffness"
- "Nice massage"

Thus, few participants reported tiredness (moderate feeling – likely due to the long duration of the experiment), neck stiffness and back ache (generated by the sitting position and weight of the head-mounted display over time).

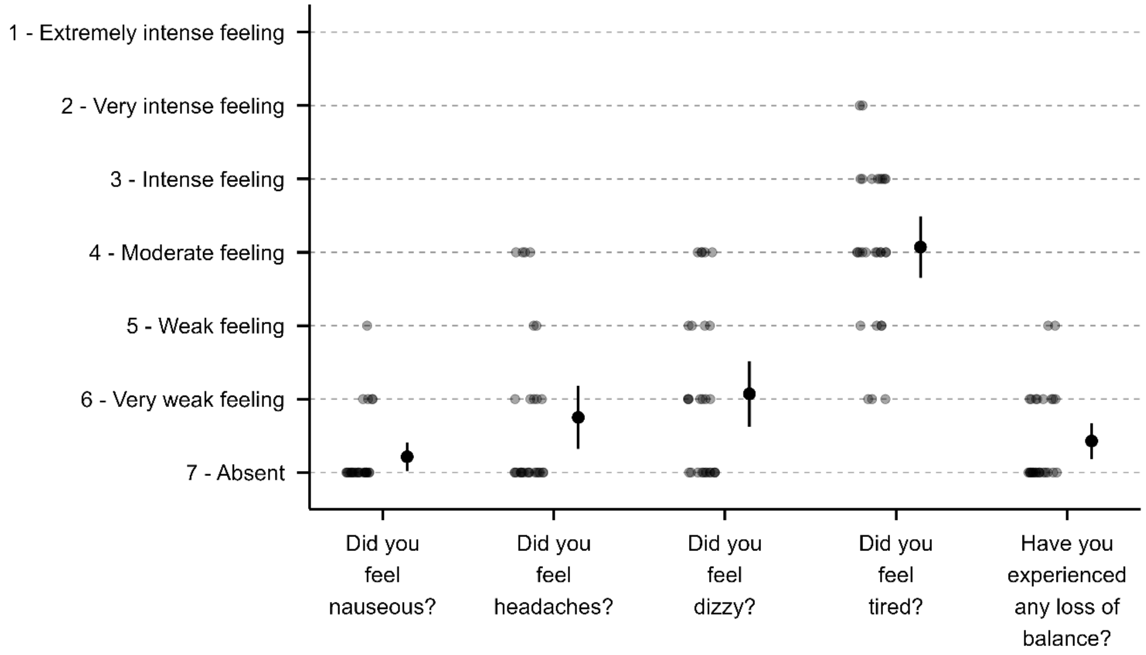

Supplementary Figure 16. Participants responses to symptoms and effects induced questionnaire reported in study 1. Each dot indicates a participant's answer. The dots with the bars on the right side indicate the in-between subject mean for each question. Error bars represent 95% confidence interval. n = 28 healthy participants. Source data are provided as a Source Data file.

Supplementary Note 9: PD-nH vs PD-PH technical specifications (study 2)

	Levene's Test for Equality of Variances		*t-test for Equality of Means*				
	F	P-value	t	df	*P-value (two-tailed)*	95% CI lower	95% CI upper
Calibration scale factor	0.087	0.77	0.83	115.53	0.41	-0.058	0.14

Supplementary Table 22. Calibration scale factor specifications (PD-nH vs PD-PH).

	Levene's Test for Equality of Variances		*t-test for Equality of Means*				
	F	P-value	t	df	*P-value (two-tailed)*	95% CI lower	95% CI upper
Refresh rate	0.85	0.36	-1.21	105.44	0.23	-8.79	2.13

Supplementary Table 23. Refresh rate specifications (PD-nH vs PD-PH).

	Levene's Test for Equality of Variances		*t-test for Equality of Means*				
	F	P-value	t	df	*P-value (two-tailed)*	95% CI lower	95% CI upper
Resolution	1.12	0.29	-0.96	96.47	0.34	-477625	165383

Supplementary Table 24. Resolution specifications (PD-nH vs PD-PH).

	PD-nH	*PD-PH*
Computer	36	43
Tablet	19	20

Supplementary Table 25. Use of a computer or tablet specifications (PD-nH vs PD-PH). Fisher's exact test for count data (p = 0.85)

	PD-nH	*PD-PH*
Windows	32	43
Mac OSX	11	11
Android	7	4
IOS	3	2
Linux	2	2
Chrome OS	0	1

Supplementary Table 26. OS family specifications (PD-nH vs PD-PH). Fisher's exact test for count data (p = 0.72)

	PD-nH	*PD-PH*
Chrome	25	24
Firefox	10	8
Safari	9	11
Edge	5	14
Mobile Safari	2	2
Samsung internet	1	3
Chrome mobile	0	1
Chrome mobile iOS	1	0
Facebook	1	0
Amazon Silk	1	0

Supplementary Table 27. Browser family specifications (PD-nH vs PD-PH). Fisher's exact test for count data (p = 0.40)

Supplementary Table 1. Statistical results for the ratings of the robot-induced sensation questionnaire (study 1)

Question	Synchronous Mean	SD	Asynchronous Mean	SD	X^2 value	df	N	Significance
I felt as if I was touching my back myself	3.00	2.13	2.04	2.01	5.78	1	28	0.016
I felt as if someone else's was touching my back	2.25	2.01	3.00	2.13	10.40	1	28	0.001
I felt as if someone was standing close to me	1.50	1.99	2.29	1.96	12.00	1	28	0.0005
I felt as if I was not controlling my movements or actions	1.14	1.53	2.61	2.10	11.20	1	28	0.0008
I felt as if someone was standing in front of me	0.57	1.14	0.50	0.75	0.04	1	28	0.83
I felt as if I had two body	0.71	1.33	0.96	1.69	1.23	1	28	0.27
I felt anxious/stressed	0.86	1.30	1.18	1.49	2.19	1	28	0.14

Supplementary Table 2. Statistical results for the general estimation performance (study 1).

One sample t-test between the presented numerosity and estimated numerosity for each stimuli type (virtual human agents or control objects) and each presented numerosity (A). Paired samples t-test for equality of means and Levene's test for equality of variance on estimated numerosity between stimuli type (virtual human agents or control objects) for each presented numerosity (B).

A)

Numerosity	Stimuli	Mean	SD	95% CI lower	95% CI upper	t value	df	*P-value (two-tailed)*
5	Humans	5.09	0.15	5.04	5.15	3.30	27	0.003
	Boxes	5.10	0.19	5.02	5.17	2.75	27	0.011
6	Humans	6.25	0.41	6.09	6.41	3.26	27	0.003
	Boxes	6.32	0.39	6.17	6.47	4.35	27	0.0002
7	Humans	7.52	0.68	7.26	7.78	4.08	27	0.0004
	Boxes	7.53	0.75	7.24	7.82	3.78	27	0.0008
8	Humans	8.72	1.12	8.29	9.16	3.41	27	0.002
	Boxes	8.78	1.05	8.38	9.19	3.95	27	0.0005

Numerosity	Stimuli	Cohen's d	95% CI lower	95% CI upper
5	Humans	0.62	0.21	1.02
	Boxes	0.52	0.12	0.91
6	Humans	0.61	0.21	1.02
	Boxes	0.82	0.39	1.25
7	Humans	0.77	0.34	1.19
	Boxes	0.71	0.29	1.13
8	Humans	0.64	0.23	1.05
	Boxes	0.75	0.32	1.16

B)

	Levene's Test for Equality of Variances		*t-test for Equality of Means*				
Numerosity	F	P-value	t	df	*P-value (two-tailed)*	95% CI lower	95% CI upper
5	2.72	0.10	-0.11	27	0.92	-0.07	0.07
6	0.23	0.64	-0.78	27	0.44	-0.25	0.11
7	0.42	0.52	-0.10	27	0.92	-0.23	0.21
8	0.0003	0.99	-0.51	27	0.62	-0.31	0.18

Supplementary Table 3: statistical results for the numerosity estimation task estimated numerosity (study 1)

ToS corresponds to the type of stimuli presented (virtual human agents or control objects). RSS corresponds to the robotic sensorimotor stimulation (synchronous or asynchronous). PN corresponds to the presented numerosity.

	Sum Sq	Mean Sq	NumDF	DenDF	F value	Pr(>F)
ToS	0.71	0.71	1	2197	1.00	0.32
RSS	0.40	0.40	1	2197	0.56	0.45
PN	4175.09	1391.70	3	2197	1945.81	0
ToS:RSS	8.26	8.26	1	2197	11.54	0.0007
ToS:PN	0.46	0.15	3	2197	0.22	0.89
RSS:PN	1.19	0.40	3	2197	0.56	0.64
ToS:RSS:PN	1.78	0.59	3	2197	0.83	0.48

Estimated marginal means and contrasts of the interaction ToS:RSS by ToS. Values or RSS are either S corresponding to synchronous robotic sensorimotor stimulation or A corresponding to asynchronous robotic sensorimotor stimulation.

ToS	RSS	emmean	SE	df	lower.CL	upper.CL
NEH	S	6.82	0.10	33.24	6.59	7.05
NEH	A	6.97	0.10	33.24	6.74	7.20
NEO	S	6.98	0.10	33.24	6.75	7.21
NEO	A	6.89	0.10	33.24	6.65	7.12

Contrast	ToS	estimate	SE	df	95% CI lower	95% CI upper	*t*	P-value
S – A	NEH	-0.15	0.05	2197	-0.25	-0.5	-2.9	0.003
S – A	NEO	0.09	0.05	2197	-0.004	0.19	1.9	0.06

Supplementary Table 4: statistical results for the numerosity estimation task response time (study 1)

ToS corresponds to the type of stimuli presented (virtual human agents or control objects). RSS corresponds to the robotic sensorimotor stimulation (synchronous or asynchronous). PN corresponds to the presented numerosity.

	Sum Sq	Mean Sq	NumDF	DenDF	F value	Pr(>F)
ToS	1.32	1.32	1	2197	0.73	0.39
RSS	5.44	5.44	1	2197	3.01	0.08
PN	77.79	25.93	3	2197	0.14	3.74e-10
ToS:RSS	0.001	0.001	1	2197	0.0008	0.98
ToS:PN	2.08	0.69	3	2197	0.38	0.76
RSS:PN	1.77	0.59	3	2197	0.33	0.81
ToS:RSS:PN	1.58	0.53	3	2197	0.29	0.83

Supplementary Table 5. Statistical results for the general estimation performance (study 2).

One sample t-test between the presented numerosity and estimated numerosity for each stimuli type (virtual human agents or control objects) and each presented numerosity (A). Paired samples t-test for equality of means and Levene's test for equality of variance on estimated numerosity between stimuli type (virtual human agents or control objects) for each presented numerosity (B).

A)

Numerosity	Condition	Mean	SD	95% CI lower	95% CI upper	t value	df	*P-value (two-tailed)*
5	Humans	6.15	0.95	5.98	6.33	13.13	117	<2.2e-16
	Boxes	5.52	0.75	5.39	5.66	7.54	117	1.1e-11
6	Humans	7.51	1.17	7.29	7.72	13.96	117	<2.2e-16
	Boxes	6.56	0.99	6.37	6.74	6.09	117	1.5e-8
7	Humans	8.79	1.72	8.48	9.10	11.32	117	<2.2e-16
	Boxes	7.56	1.28	7.33	7.80	4.79	117	5.0e-6
8	Humans	10.42	2.51	9.96	10.88	10.48	117	<2.2e-16
	Boxes	8.47	1.61	8.18	8.76	3.17	117	0.002

Numerosity	Stimuli	Cohen's d	95% CI lower	95% CI upper
5	Humans	1.21	0.97	1.45
	Boxes	0.69	0.49	0.89
6	Humans	1.28	1.04	1.53
	Boxes	0.56	0.37	0.75
7	Humans	1.04	0.82	1.27
	Boxes	0.44	0.25	0.63
8	Humans	0.96	0.74	1.18
	Boxes	0.29	0.11	0.48

B)

	Levene's Test for Equality of Variances		*t-test for Equality of Means*				
**Numerosity**	**F**	**P-value**	**t**	**df**	***P-value (two-tailed)***	**95% CI lower**	**95% CI upper**
5	6.06	0.15	9.17	117	1.9e-15	0.49	0.77
6	5.89	0.016	9.83	117	<2.2e-16	0.76	1.14
7	8.29	0.004	9.80	117	<2.2e-16	0.98	1.48
8	17.56	3.9e-5	11.34	117	<2.2e-16	1.61	2.29

Supplementary Table 6: self-report questionnaire on alteration of perception (study 2)

Participant ID	*Visual disturbance*
Passage hallucination	Have you ever felt that someone, something or a shadow passing or moving on your side(s)?
Presence hallucination	Have you ever felt that someone is behind you or close to you when there is actually no one there?
Visual illusion	Have you ever seen something else instead of a real object or living thing? For example, seeing an animal instead of a bush, a stain on the floor transforms into a crawling insect, or stationary objects perceived as being in motion.
Visual hallucination	Have you ever seen objects, people, animals or scenes that others could not see and claim not to be real?

Supplementary Table 7: visual disturbances reported by PD patients that were rejected for the numerosity estimation task analysis (study 2)

Participant ID	*Visual disturbance*
pnj1_kxdax5e0-112a35c7-5746-66d1-cfdb-fa578961d8ca	“Double vision, occasional hallucination”
pnj1_ky2stwm4-88cc31f9-847e-61f1-ef9b-39b5de3f2251	“yes . Things change colour and appearance”
pnj1_kswwqp-eb784aee-b97b-2a9d-d051-16ff676a8585	« mes paupilles deviennent lourdes et je n'arrive pas à garder les yeux ouverts et j'ai difficile à rouvrir les yeux »
pnj1_kx67m1i3-1e0ff548-715f-c096-cecd-41e386659a9d	“Yes, I see images of my children in places where they are not present. I see quick images running with the corner of my eyes. I see shoes walking on the floor »
pnj1_kx8x13l7-bbfc5884-8005-c487-db78-9ad240b838da	« Double vision »
pnj1_l140oidg-727cee41-8f28-a9bc-2e29-8c2ec7677c8e	« oui. Impression de flou, de voir à travers de l'eau »
pnj1_l1g3lle8-b279f7f7-9ca7-c886-5dc7-7d2818cb46aa	« voie double par moment »
pnj1_l2kxn2kf-3356f4f5-5ffe-f74d-ea29-b23a689e4a65	« diplopie »
pnj1_l2puopo1-e92db4cb-614b-6f4c-95ca-5d56f98dd084	« Troubles d'accommodation, diplopie, carré noir »
pnj1_l3elk8ml-3a8cb639-341d-aec7-f1f9-45678f6b4734	“shadows seen in peripheral vision”

Supplementary Table 8: Occurrences of the different types of experienced hallucinations (study 2)

Participants reported the occurrence as Never, Rarely (less than once a month), Occasionally (several times, but less than once a week), Frequently (several times a week, but less than once a day), or Daily (almost every day, several times a day)

A) Table with all participants (N=170)

	Passage sensation	*Presence hallucination*	*Visual Illusion*	*Visual hallucinations*
Never	80	101	110	138
Rarely	45	30	33	19
Occasionally	25	25	17	8
Frequently	16	10	4	5
Daily	4	4	6	0

B) Table with only the participants kept for numerosity estimation data analysis (N=118).

	Passage sensation	*Presence hallucination*	*Visual Illusion*	*Visual hallucinations*
Never	62	55	84	96
Rarely	20	26	18	13
Occasionally	19	23	10	6
Frequently	13	10	3	3
Daily	4	4	3	0

Supplementary Table 9: statistical results for the numerosity estimation task estimated numerosity (study 2)

ToS corresponds to the type of stimuli presented (virtual human agents or control objects). PDG corresponds to the PD group (PD-nH or PD-PH). PN corresponds to the presented numerosity.

	Sum Sq	Mean Sq	NumDF	DenDF	F value	Pr(>F)
ToS	3140.26	3140.26	1	9084.91	1188.26	7.9e-245
PDG	10.68	10.68	1	115.88	4.04	0.047
PN	16293.09	5431.03	3	9084.31	2055.08	0
ToS:PDG	142.22	142.22	1	9084.91	53.81	2.4e-13
ToS:PN	524.67	174.89	3	9084.05	66.18	2.5e-42
PDG:PN	132.50	44.17	3	9084.31	16.71	8.0e-11
ToS:RSS:PN	41.86	13.95	3	9084.05	5.28	0.001

Estimated marginal means and contrasts of the interaction ToS:PDG by ToS.

ToS	PDG	emmean	SE	df	lower.CL	upper.CL
NEH	PD-nH	7.86	0.15	122.13	7.51	8.21
NEH	PD-PH	8.53	0.14	122.19	8.20	8.86
NEO	PD-nH	6.94	0.15	122.33	6.59	7.29
NEO	PD-PH	7.11	0.14	122.13	6.78	7.44

Contrast	ToS	estimate	SE	df	95% CI lower	95% CI upper	*t*	P-value
PD-nH – PD-PH	NEH	-0.67	0.21	122.16	-1.09	-0.25	-3.16	0.002
PD-nH – PD-PH	NEO	-0.17	0.21	122.24	-0.59	0.25	-0.81	0.42

Supplementary Table 10: statistical results for the numerosity estimation task estimated numerosity with covariates (study 2)

ToS corresponds to the type of stimuli presented (virtual human agents or control objects). PDG corresponds to the PD group (PD-nH or PD-PH). PN corresponds to the presented numerosity.

	Sum Sq	Mean Sq	NumDF	DenDF	F value	Pr(>F)
ToS	3140.53	3140.53	1	9084.96	1188.36	7.5e-245
PDG	15.34	15.34	1	109.92	5.81	0.018
PN	16293.83	5431.28	3	9084.31	2055.17	0
PD_side	7.51	2.50	3	109.96	0.95	0.42
PD_duration	7.36	7.36	1	109.95	2.79	0.098
age	1.73	1.73	1	109.98	0.66	0.42
gender	6.08	6.08	1	109.92	2.30	0.13
ToS:PDG	142.12	142.12	1	9084.90	53.78	2.4e-13
ToS:PN	524.57	174.86	3	9084.07	66.16	2.6e-42
PDG:PN	132.41	44.14	3	9084.32	16.70	8.1e-11
ToS:RSS:PN	41.87	13.96	3	9084.07	5.28	0.001

Estimated marginal means and contrasts of the interaction ToS:PDG by ToS.

ToS	PDG	emmean	SE	df	lower.CL	upper.CL
NEH	PD-nH	7.75	0.18	114.36	7.35	8.16
NEH	PD-PH	8.52	0.18	113.94	8.12	8.92
NEO	PD-nH	6.83	0.18	114.49	6.43	7.24
NEO	PD-PH	7.10	0.18	113.98	6.70	7.50

Contrast	ToS	estimate	SE	df	95% CI lower	95% CI upper	*t*	P-value
PD-nH – PD-PH	NEH	-0.76	0.22	115.60	-1.19	-0.34	-3.53	0.0006
PD-nH – PD-PH	NEO	-0.27	0.22	115.70	-0.69	0.16	-1.23	0.22

Supplementary Table 11: statistical results for the numerosity estimation task response time (study 2)

ToS corresponds to the type of stimuli presented (virtual human agents or control objects). PDG corresponds to the PD group (PD-nH or PD-PH). PN corresponds to the presented numerosity.

	Sum Sq	Mean Sq	NumDF	DenDF	F value	Pr(>F)
ToS	199348501.1	199348501.1	1	9084.09	78.67	8.7e-19
PDG	1463771.6	1463771.6	1	115.76	0.58	0.45
PN	379889853.3	126629951.1	3	9083.90	49.98	5.0e-32
ToS:PDG	965158.8	965158.8	1	9084.09	0.38	0.54
ToS:PN	866669.4	288889.8	3	9083.81	0.11	0.95
PDG:PN	16896421.8	5632140.6	3	9083.90	2.22	0.08
ToS:RSS:PN	2195005.4	731668.5	3	9083.81	0.29	0.83

Estimated marginal means of ToS at each numerosity

ToS	emmean	SE	df	lower.CL	upper.CL
NEH	4617.16	181.23	117.97	4258.29	4976.04
NEO	4322.05	181.23	117.99	3963.15	4680.94

Numerosity	Condition	Emmean	SE	df	95% CI lower	95% CI upper
5	Humans	4295.843	185.7081	130.0746	3874.723	4716.963
	Boxes	4030.099	185.8071	130.3516	3608.765	4451.433
6	Humans	4589.319	185.7256	130.1235	4168.161	5010.476
	Boxes	4276.233	185.7261	130.1248	3855.074	4697.392
7	Humans	4720.750	185.7494	130.1900	4299.541	5141.959
	Boxes	4408.486	185.7673	130.2401	3987.238	4829.734
8	Humans	4862.735	185.7548	130.2053	4441.514	5283.956
	Boxes	4573.365	185.7606	130.2214	4152.131	4994.598

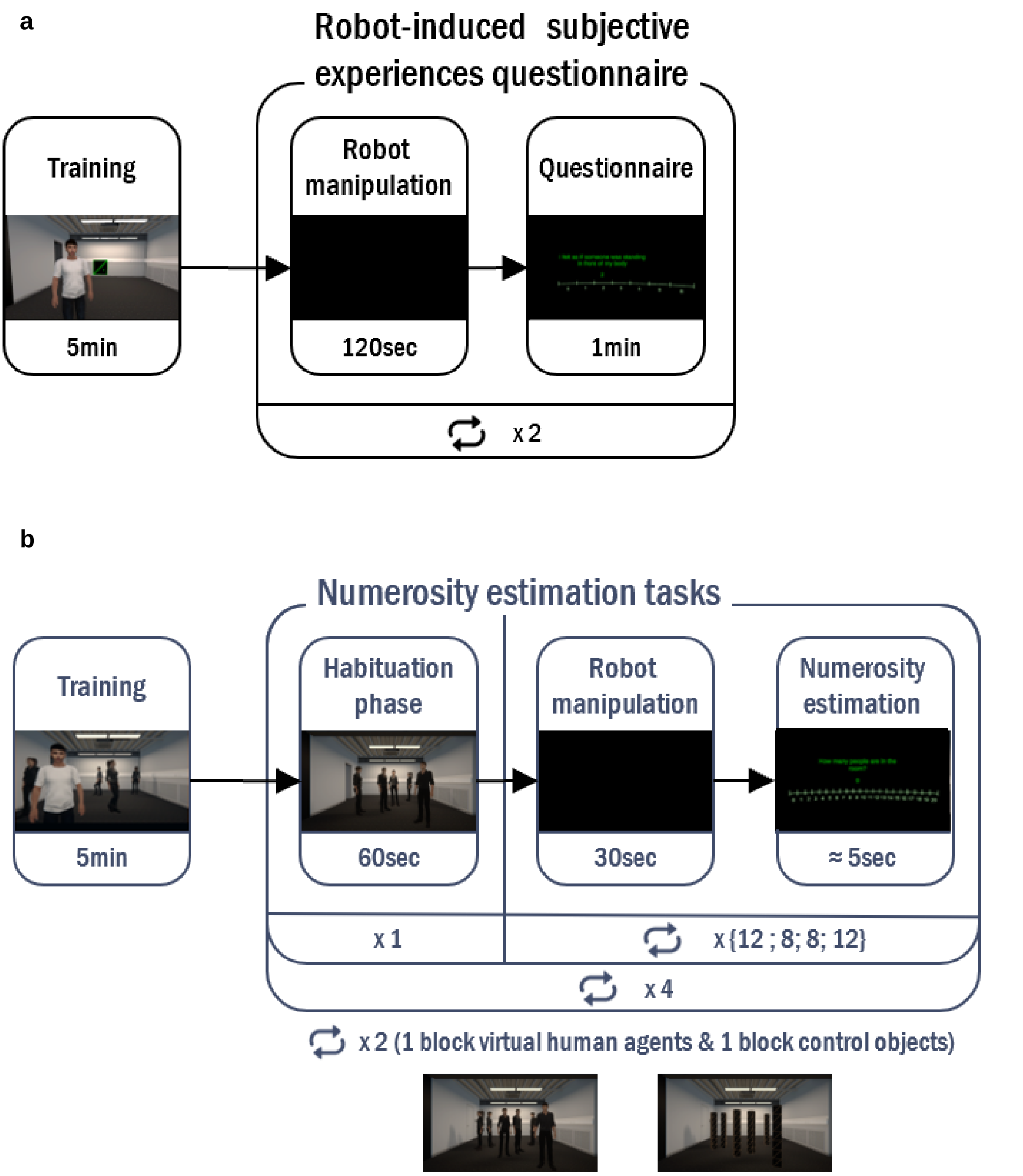

Supplementary Figure 1. Experimental protocol (study 1). Schematic representation of the experimental protocol of the a) robot-induced subjective experiences questionnaire and b) human and object numerosity estimation tasks (study 1). A detailed view of a numerosity trial (sensorimotor robotic manipulation followed by numerosity estimation) is depicted in Figure 1

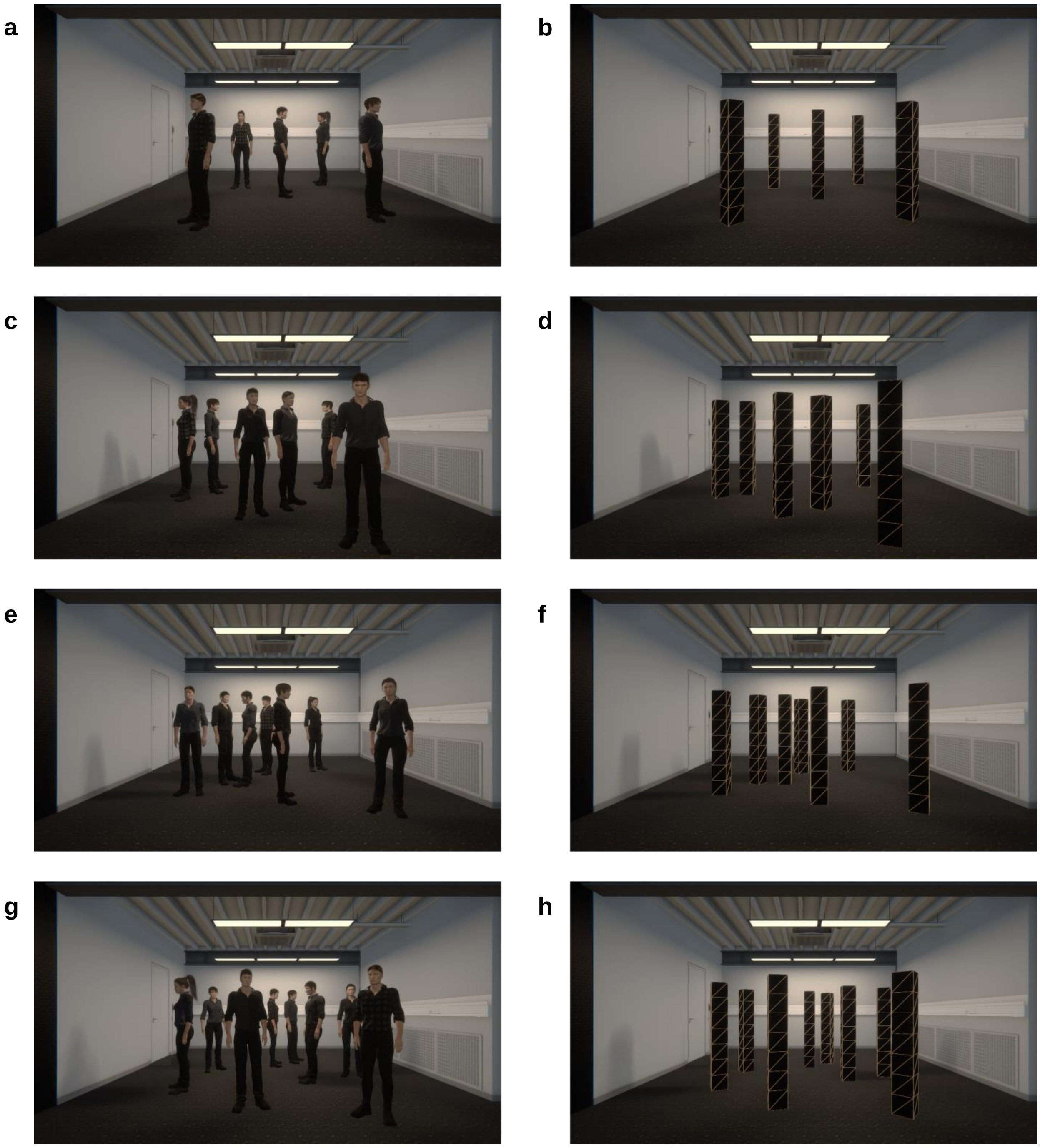

Supplementary Figure 2. Example of human and object numerosity estimation tasks stimuli. Example with (a) five, (c) six, (e) seven, (g) eight virtual human agents (human numerosity estimation task) or (b) five, (d) six, (f) seven, (h) eight objects (object numerosity estimation task).

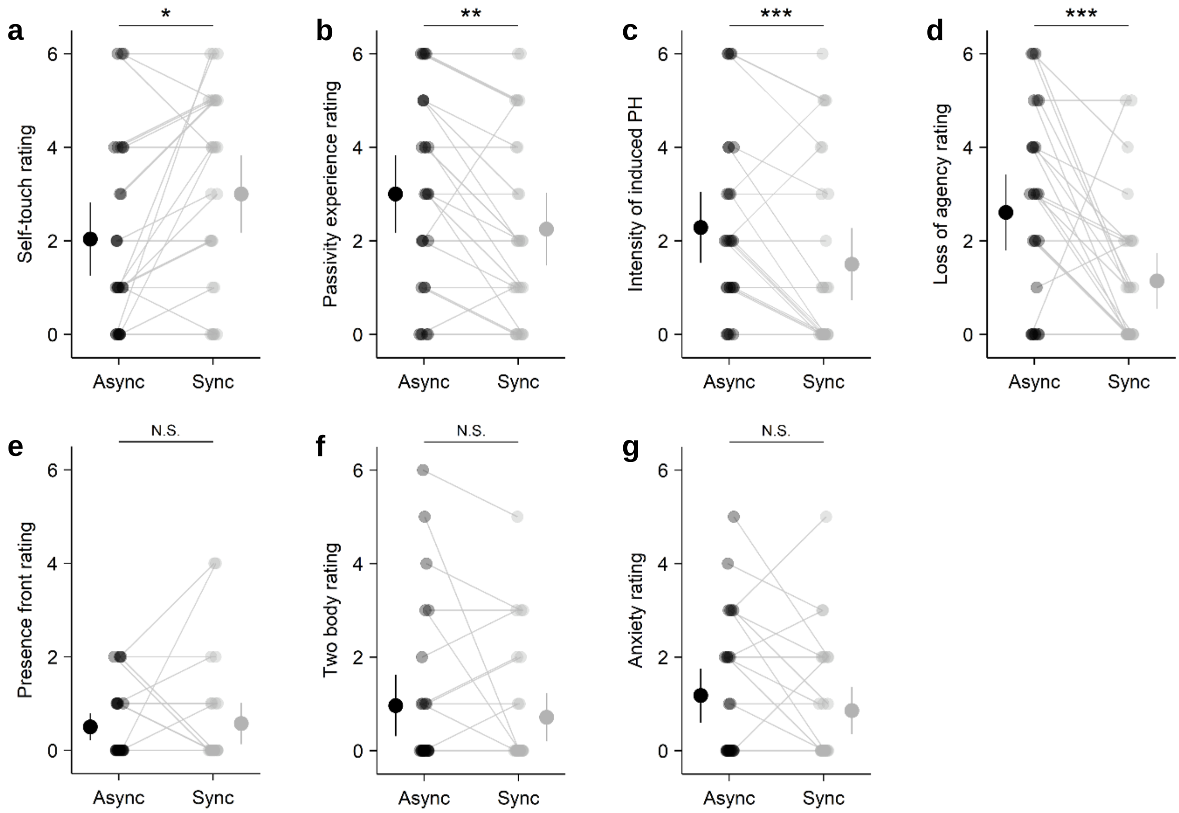

Supplementary Figure 3. Robot induced sensation questionnaire ratings (asynchronous versus synchronous) (study 1). (a) Self-touch assessment ratings. (b) Passivity experience assessment ratings. (c) robot-induced presence hallucination assessment ratings. (d) Loss of agency assessment ratings. (e) Presence on the front assessment ratings. (f) Two-body assessment ratings. (g) Anxiety assessment ratings. Each linked pair of dots indicates the individual rating of the intensity of the assessed sensation (Asynchronous condition (dark grey) and synchronous condition (light grey)). The dots with the bar on the left and right sides indicate the mixed-effects linear regression between asynchronous (dark grey) and synchronous (light gray) sensorimotor stimulation. Error bar represents 95% confidence interval. *P ≤ 0.05 ; **P ≤ 0.01 ; ***P ≤ 0.001. n = 28 healthy participants. Source data are provided as a Source Data file.

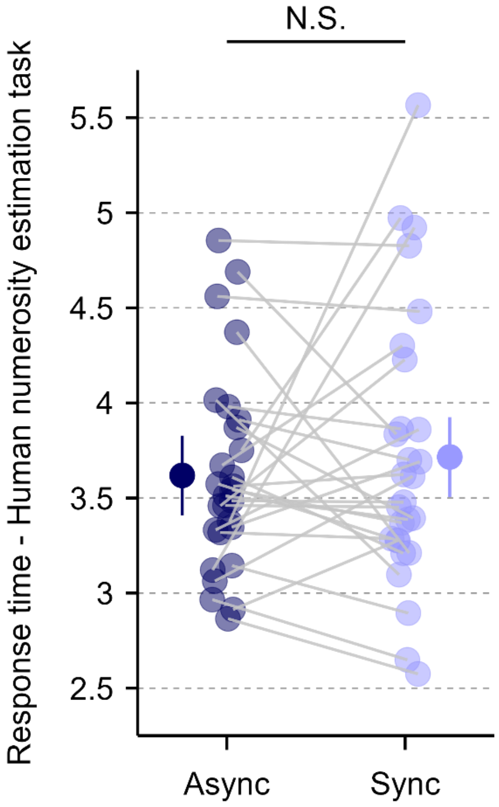

Supplementary Figure 4. Human numerosity estimation task response time (synchronous, asynchronous) (study 1). Each linked pair of dots indicates the individual human numerosity estimation task response time mean estimate in asynchronous (dark blue) and synchronous (light blue) sensorimotor stimulation. The dots with the bar on the left and right sides indicate the mixed-effects linear regression between asynchronous (dark blue) and synchronous (light blue) sensorimotor stimulation. Response time is expressed in seconds. Error bar represents 95% confidence interval. N.S., not significant. n = 28 healthy participants. Source data are provided as a Source Data file.

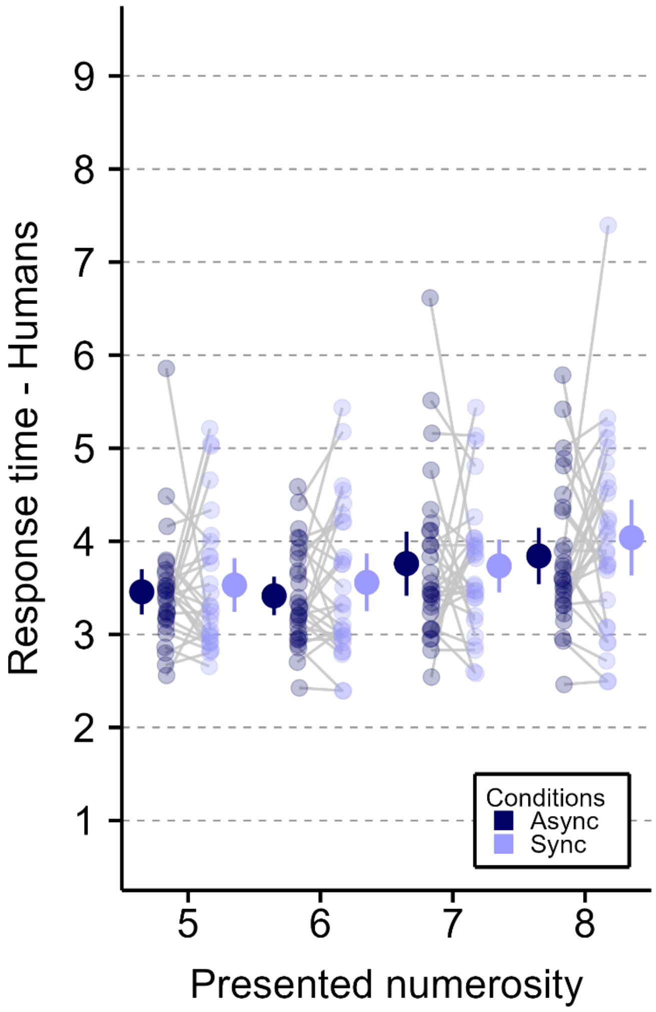

Supplementary Figure 5. Human numerosity estimation task response time (synchronous, asynchronous) (study 1). Task response time is shown for each presented numerosity in the human numerosity estimation task, separately for the asynchronous and synchronous sensorimotor stimulation. Each linked pair of dots indicates the individual human numerosity estimation task response time mean estimate at each presented numerosity in asynchronous (dark blue) and synchronous (light blue) sensorimotor stimulation. The dots with the bar on the left and right sides indicate the mixed-effects linear regression between asynchronous (dark blue) and synchronous (light blue) sensorimotor stimulation at each presented numerosity. Response time is expressed in seconds. Error bar represents 95%

confidence interval. n = 28 healthy participants. Source data are provided as a Source Data file.

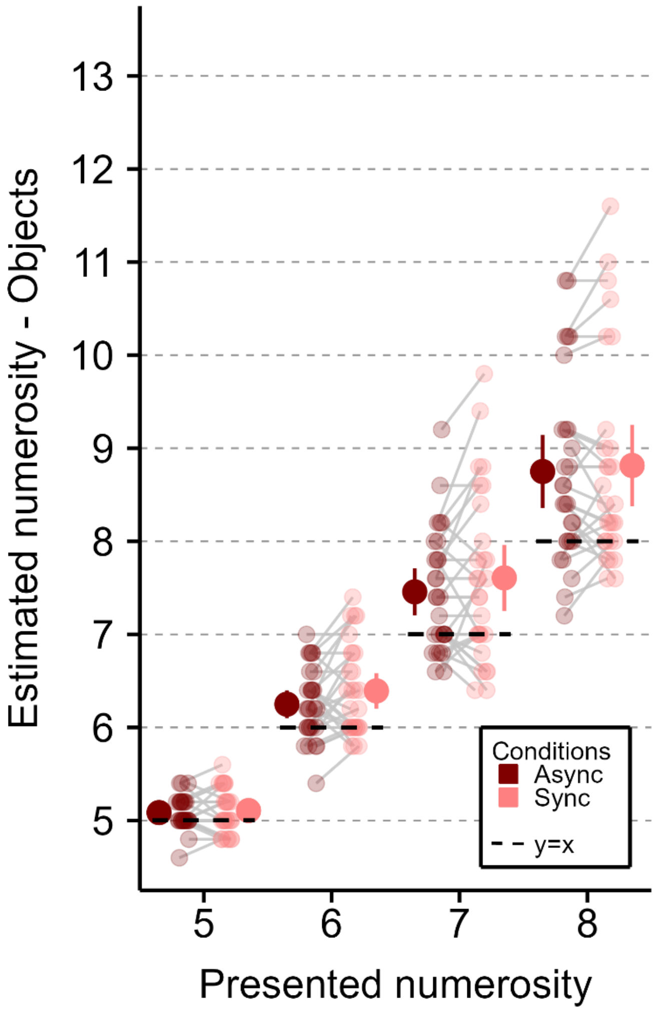

Supplementary Figure 6. Object numerosity estimation task (each presented numerosity, synchronous, asynchronous) (study 1). Task performance is shown for each presented numerosity in the object numerosity estimation task, separately for the asynchronous and synchronous sensorimotor stimulation. Each linked pair of dots indicates the individual object numerosity estimation task mean estimate at the corresponding presented numerosity in asynchronous (dark red) and synchronous (light red) sensorimotor stimulation. The dots with the bar on the left and right sides indicate the mixed-effects linear regression between asynchronous (dark red) and synchronous (light red) sensorimotor stimulation at each presented numerosity. Error bar represents 95% confidence interval. n = 28 healthy participants. Source data are provided as a Source Data file.

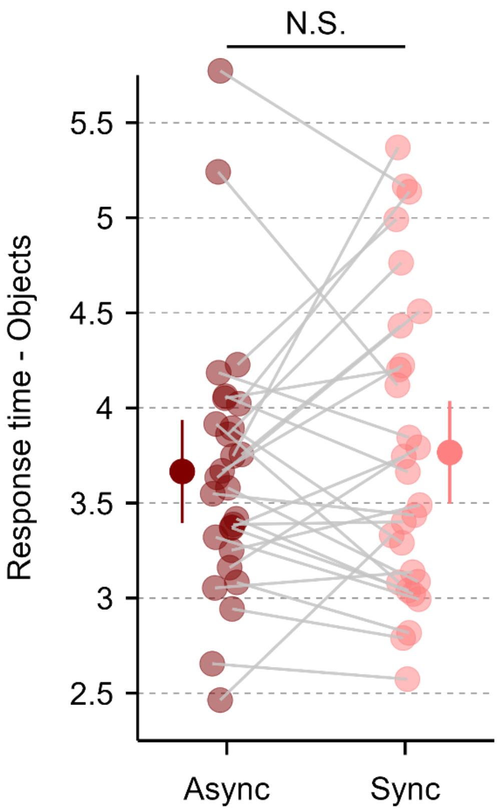

Supplementary Figure 7. Object numerosity estimation task response time (synchronous, asynchronous) (study 1). Each linked pair of dots indicates the individual object numerosity estimation task response time mean estimate in asynchronous (dark red) and synchronous (light red) sensorimotor stimulation. The dots with the bar on the left and right sides indicate the mixed-effects linear regression between asynchronous (dark red) and synchronous (light red) sensorimotor stimulation. Response time is expressed in seconds. Error bar represents 95% confidence interval. N.S., not significant. n = 28 healthy participants. Source data are provided as a Source Data file.

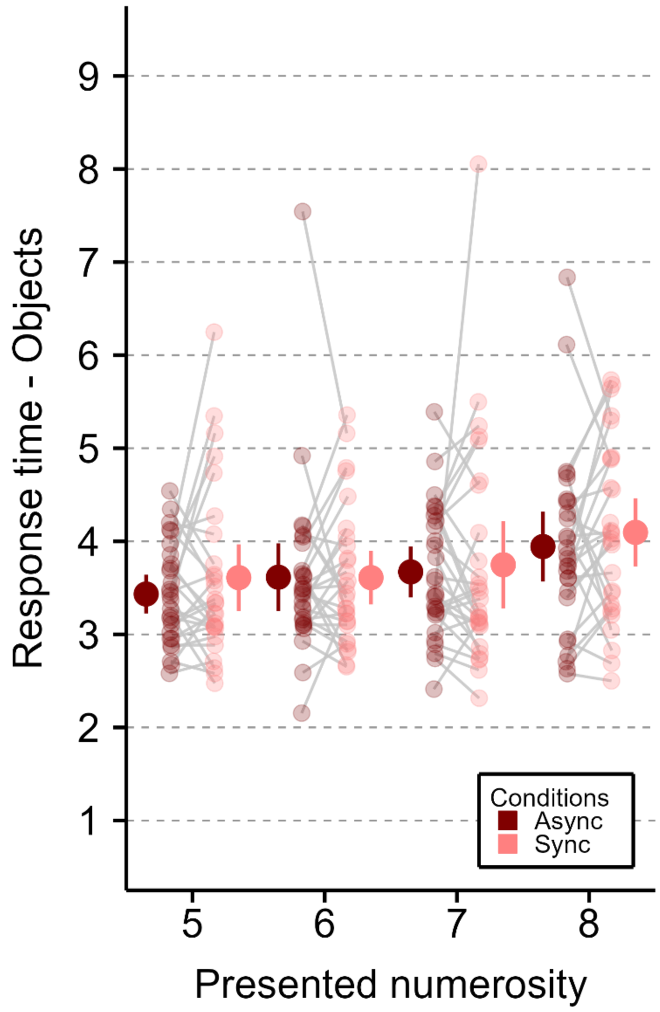

Supplementary Figure 8. Object numerosity estimation task response time (synchronous, asynchronous) (study 1). Task response time is shown for each presented numerosity in the object numerosity estimation task, separately for the asynchronous and synchronous sensorimotor stimulation. Each linked pair of dots indicates the individual object numerosity estimation task response time mean estimate at each presented numerosity in asynchronous (dark red) and synchronous (light red) sensorimotor stimulation. The dots with the bar on the left and right sides indicate the mixed-effects linear regression between asynchronous (dark red) and synchronous (light red) sensorimotor stimulation at each presented numerosity. Response time is expressed in seconds. Error bar represents 95%

confidence interval. n = 28 healthy participants. Source data are provided as a Source Data file.

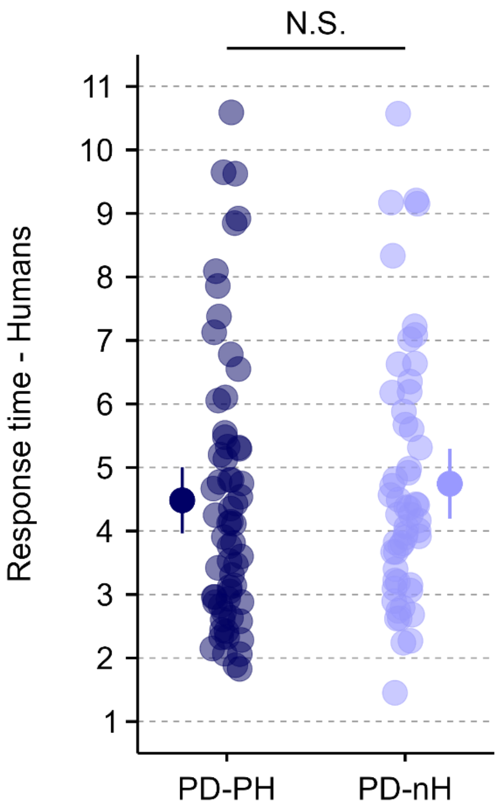

Supplementary Figure 9. Human numerosity estimation task response time in PD patients (PD-PH and PD-nH) (study 2). Each dot indicates the individual human numerosity estimation task response time mean estimate (PD-PH (dark blue) and PD-nH (light blue)). The dots with the bar on the left and right sides indicate the mixed-effects linear regression between PD-PH (dark blue) and PD-nH (light blue). Response time is expressed in seconds. Error bar represents 95% confidence interval. N.S., not significant. n = 118 patients with PD (63 PD-PH & 55 PD-nH). Source data are provided as a Source Data file. PD = Parkinson's Disease; PD-PH = Parkinson's Disease patients with Presence Hallucination; PD-nH = Parkinson's Disease patients with no Hallucination.

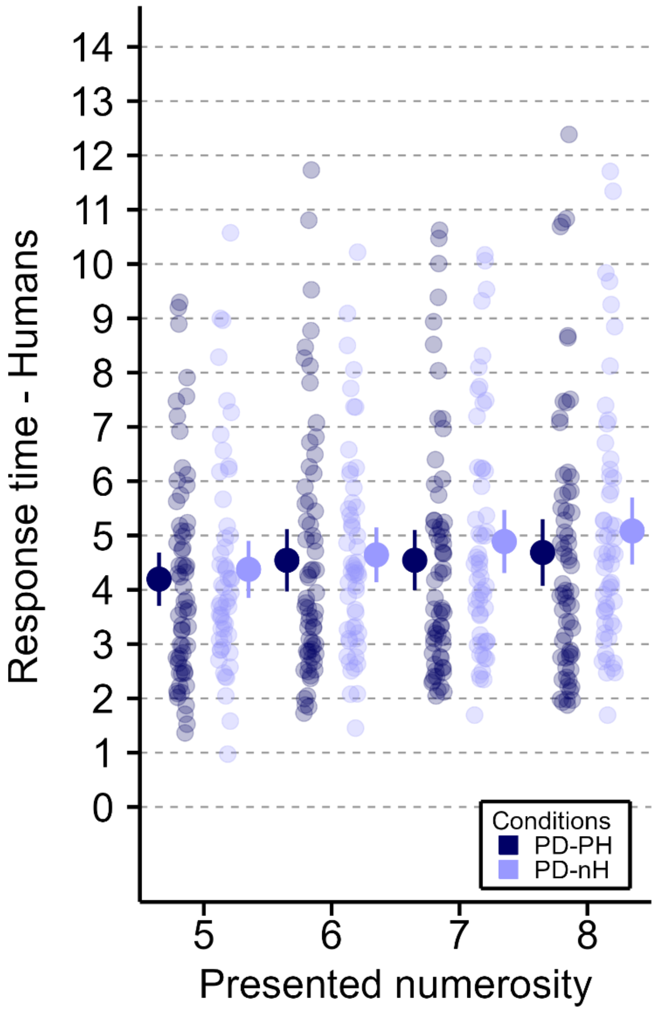

Supplementary Figure 10. Human numerosity estimation task response time in PD patients (PD-PH and PD-nH) (study 2). Response time is shown in PD patients for each tested numerosity in the human numerosity estimation task for PD-PH and PD-nH separately. Each dot indicates the individual human numerosity estimation task response time mean estimate at the corresponding presented numerosity (PD-PH (dark blue) and PD-nH (light blue)). The dots with the bar on the left and right sides indicate the mixed-effects linear regression between PD-PH (dark blue) and PD-nH (light blue) at each presented numerosity. Response time is expressed in seconds. Error bar represents 95% confidence interval. n = 118 patients with PD (63 PD-PH & 55 PD-nH). Source data are provided as a Source Data

file. PD = Parkinson's Disease; PD-PH = Parkinson's Disease patients with Presence Hallucination; PD-nH = Parkinson's Disease patients with no Hallucination.

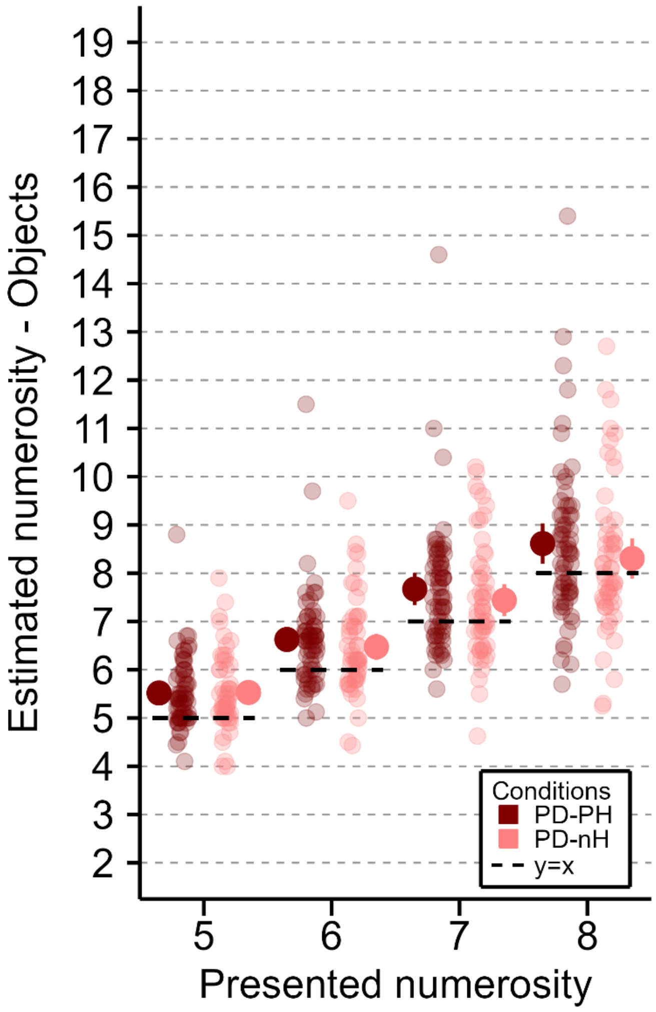

Supplementary Figure 11. Object numerosity estimation task in PD patients (each presented numerosity, PD-PH vs PD-nH) (study 2). Performance is shown in PD patients for each tested numerosity in the object numerosity estimation task for PD-PH and PD-nH separately. Each dot indicates the individual object numerosity estimation task mean estimate at the corresponding presented numerosity (PD-PH (dark red) and PD-nH (light red)). The dots with the bar on the left and right sides indicate the mixed-effects linear regression between PD-PH (dark red) and PD-nH (light red) at each presented numerosity. Error bar represents 95% confidence interval. n = 118 patients with PD (63 PD-PH & 55 PD-nH). Source data are provided as a Source Data file. PD = Parkinson's Disease; PD-PH = Parkinson's

Disease patients with Presence Hallucination; PD-nH = Parkinson's Disease patients with no Hallucination.

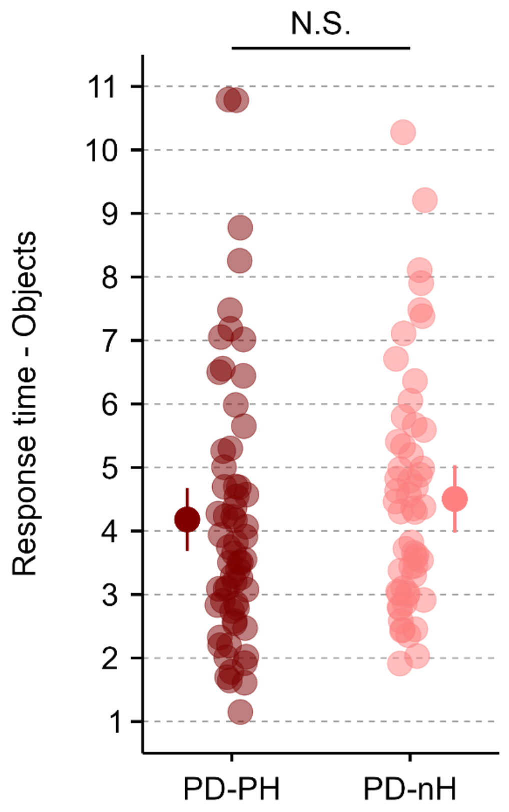

Supplementary Figure 12. Object numerosity estimation task response time in PD patients (PD-PH and PD-nH) (study 2). Each dot indicates the individual object numerosity estimation task response time mean estimate (PD-PH (dark red) and PD-nH (light red)). The dots with the bar on the left and right sides indicate the mixed-effects linear regression between PD-PH (dark red) and PD-nH (light red). Response time is expressed in seconds. Error bar represents 95% confidence interval. N.S., not significant. n = 118 patients with PD (63 PD-PH & 55 PD-nH). Source data are provided as a Source Data file. PD = Parkinson's Disease; PD-PH = Parkinson's Disease patients with Presence Hallucination; PD-nH = Parkinson's Disease patients with no Hallucination.

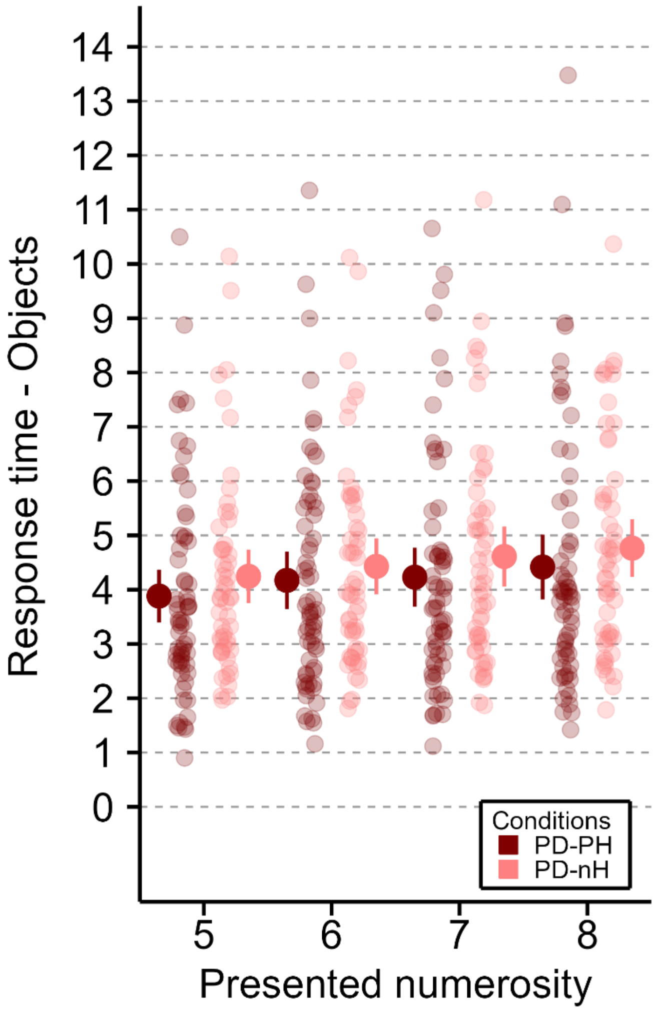

Supplementary Figure 13. Object numerosity estimation task response time in PD patients (each presented numerosity, PD-PH vs PD-nH) (study 2). Response time is shown in PD patients for each tested numerosity in the object numerosity estimation task for PD-PH and PD-nH separately. Each dot indicates the individual object numerosity estimation task response time mean estimate at the corresponding presented numerosity (PD-PH (dark red) and PD-nH (light red)). The dots with the bar on the left and right sides indicate the mixed-effects linear regression between PD-PH (dark red) and PD-nH (light red) at each presented numerosity. Response time is expressed in seconds. Error bar represents 95% confidence interval. n = 118 patients with PD (63 PD-PH & 55 PD-nH). Source data are provided as a Source Data file. PD = Parkinson's Disease; PD-PH = Parkinson's Disease patients with Presence Hallucination; PD-nH = Parkinson's Disease patients with no Hallucination.

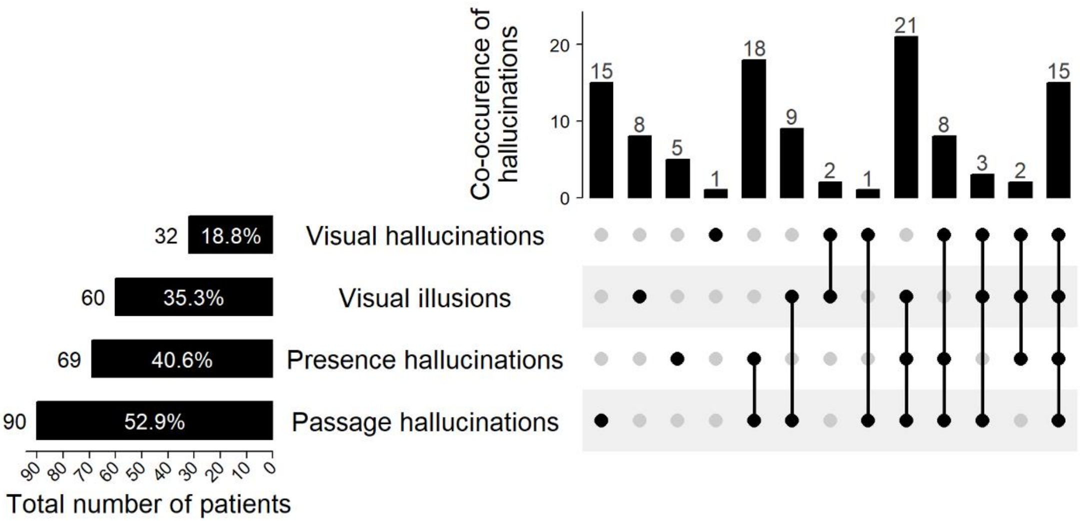

Supplementary Figure 14. Prevalence of hallucinations (study 2). Prevalence of hallucination in PD (n = 170 patients with PD). The sum of patients experiencing a specific hallucination is represented by the left bar plot. Every possible combination of multiple hallucination for a single patient is represented by the connected lines, and the number of patients is shown by the top bar plot. PD = Parkinson’s Disease.

Supplementary references

1. Morris, C. G., Press, A. & Morris, C. W. *Academic Press Dictionary of Science and Technology*. (Gulf Professional Publishing, 1992).
2. Anstis, S. M. A chart demonstrating variations in acuity with retinal position. *Vision Res.* **14**, 589–592 (1974).
3. Leibovich-Raveh, T., Lewis, D. J., Kadhim, S. A.-R. & Ansari, D. A New Method for Calculating Individual Subitizing Ranges. *J. Numer. Cogn.* **4**, 429–447 (2018).
4. Revkin, S. K., Piazza, M., Izard, V., Cohen, L. & Dehaene, S. Does Subitizing Reflect Numerical Estimation? *Psychol. Sci.* **19**, 607–614 (2008).
5. Marquardt, D. W. An Algorithm for Least-Squares Estimation of Nonlinear Parameters. *J. Soc. Ind. Appl. Math.* **11**, 431–441 (1963).
6. Bates, D., Mächler, M., Bolker, B. & Walker, S. Fitting Linear Mixed-Effects Models Using lme4. *J. Stat. Softw.* **67**, 1–48 (2015).
7. Kuznetsova, A., Brockhoff, P. B. & Christensen, R. H. B. lmerTest Package: Tests in Linear Mixed Effects Models. *J. Stat. Softw.* **82**, 1–26 (2017).
8. Team, R. C. R: A language and environment for statistical computing. (2013).
9. Kaufman, E. L., Lord, M. W., Reese, T. W. & Volkmann, J. The Discrimination of Visual Number. *Am. J. Psychol.* **62**, 498–525 (1949).
10. Chang, A. & Fox, S. H. Psychosis in Parkinson's Disease: Epidemiology, Pathophysiology, and Management. *Drugs* **76**, 1093–1118 (2016).
11. Fénelon, G. & Alves, G. Epidemiology of psychosis in Parkinson's disease. *J. Neurol. Sci.* **289**, 12–17 (2010).
12. Pacchetti, C. *et al.* Relationship between hallucinations, delusions, and rapid eye movement sleep behavior disorder in Parkinson's disease. *Mov. Disord.* **20**, 1439–1448 (2005).

13. Fénelon, G., Soulas, T., De Langavant, L. C., Trinkler, I. & Bachoud-Lévi, A.-C. Feeling of presence in Parkinson's disease. *J. Neurol. Neurosurg. Psychiatry* **82**, 1219–1224 (2011).

14. Pagonabarraga, J. *et al.* Minor hallucinations occur in drug-naive Parkinson's disease patients, even from the premotor phase. *Mov. Disord.* **31**, 45–52 (2016).
