# Supplementary material for: Numerosity estimation of virtual humans as a digital-robotic marker for hallucinations in Parkinson’s disease
Source: Nat Commun. 2024 Mar 12;15:1905. doi: 10.1038/s41467-024-45912-w (PMC10933269; doi:10.1038/s41467-024-45912-w)
Supplement: Supplementary file 3 — Description of Additional Supplementary Files [file 41467_2024_45912_MOESM3_ESM.pdf]

Description of Additional Supplementary Files

File Name: Supplementary Movie 1
Description: Human numerosity estimation task (study 1) (synchronous sensorimotor robotic stimulation – control condition). Example of a human numerosity estimation task trial following synchronous sensorimotor robotic stimulation, displayed from an external view of the participant and a synchronized VR view. In each human numerosity estimation task trial, participants first manipulated the robotic system for 30 seconds (either in the asynchronous (500ms delay; presence hallucination inducing condition) or the synchronous condition (0ms delay)). This was followed by the appearance of a fixation cross (500-1500ms), indicating to participants that they could stop moving the robotic system. Then, a scene containing a different number of people (range 5 -8) was shown for 200ms and participants had to estimate the number of people they saw. This video was not recorded while a participant performed the experiment, but recorded and edited during a session dedicated to making videos to illustrate the experiment. The person portrayed is the first author of the manuscript, Mr. Albert Louis Philippe, who gave his consent for his face to be unblurred and his voice to be unaltered in the video.

File Name: Supplementary Movie 2
Description: Human numerosity estimation task (study 1) (asynchronous sensorimotor robotic stimulation – presence hallucination inducing condition). Example of a human numerosity estimation task trial following asynchronous sensorimotor robotic stimulation, displayed from an external view of the participant and a synchronized VR view. In each human numerosity estimation task trial, participants first manipulated the robotic system for 30 seconds (either in the asynchronous (500ms delay; presence hallucination inducing condition) or the synchronous condition (0ms delay)). This was followed by the appearance of a fixation cross (500 -1500ms), indicating to participants that they could stop moving the robotic system. Then, a scene containing a different number of people (range 5 -8) was shown for 200ms and participants had to estimate the number of people they saw. This video was not recorded while a participant performed the experiment, but recorded and edited during a session dedicated to making videos to illustrate the experiment. The person portrayed is the first author of the manuscript, Mr. Albert Louis Philippe, who gave his consent for his face to be unblurred and his voice to be unaltered in the video.

File Name: Supplementary Movie 3
Description: Habituation phases of a participant in VR (study 1) (human numerosity estimation task). Example of a participant during the habituation phases of the human numerosity estimation task, displayed from an external view of the participant and a synchronized VR view. This video was not recorded while a participant performed the experiment, but recorded and edited during a session dedicated to making videos to illustrate the experiment. The person portrayed is the first author of the manuscript, Mr. Albert Louis

Philippe, who gave his consent for his face to be unblurred and his voice to be unaltered in the video.

File Name: Supplementary Movie 4
Description: Human and object habituation phases comparisons (study 1) (fixed view in position of the participant and top view). The four human habituation phases and the four object habituation phases are displayed side by side for comparison, from a virtual fixed view in position of the participant and a virtual top view.

File Name: Supplementary Movie 5
Description: Robot induced sensation questionnaire training (study 1) (fixed view in position of the participant). Training of the robot induced sensation questionnaire, displayed from a virtual fixed view in position of the participant. Participants are first introduced to the virtual laboratory by the virtual experimenter, followed by training in the use of the sensorimotor robotic device, followed by training in the response mechanism (head movement and voice recognition, see Methods).

File Name: Supplementary Movie 6
Description: Human numerosity estimation task training (study 1) (fixed view in position of the participant). Training of the human numerosity estimation task, displayed from a virtual fixed view in position of the participant. The task starts with a habituation phase, during which several virtual human agents moved and discussed in the virtual environment. Then, in each human numerosity estimation task trial, participants first manipulated the robotic system for 30 seconds (either in the asynchronous (500ms delay; presence hallucination inducing condition) or the synchronous condition (0ms delay)). This was followed by the appearance of a fixation cross (500 -1500ms), indicating to participants that they could stop moving the robotic system. Then, a scene containing a different number of people (range 5 -8) was shown for 200ms and participants had to estimate the number of people they saw.

File Name: Supplementary Movie 7
Description: Object numerosity estimation task training (study 1) (fixed view in position of the participant). Training of the object numerosity estimation task, displayed from a virtual fixed view in position of the participant. The task starts with a habituation phase, during which several objects materialized and dematerialized in the virtual environment. Then, in each object numerosity estimation task trial, participants first manipulated the robotic system for 30 seconds (either in the asynchronous (500ms delay; presence hallucination inducing condition) or the synchronous condition (0ms delay)). This was followed by the appearance of a fixation cross (500 -1500ms), indicating to participants that they could stop moving the robotic system. Then, a scene containing a different number of objects (range 5-8) was shown for 200ms and participants had to estimate the number of objects they saw.
