# Supplementary material for: Numerosity estimation of virtual humans as a digital-robotic marker for hallucinations in Parkinson’s disease
Source: Nat Commun. 2024 Mar 12;15:1905. doi: 10.1038/s41467-024-45912-w (PMC10933269; doi:10.1038/s41467-024-45912-w)
Supplement: Supplementary file 11 — Reporting Summary [file 41467_2024_45912_MOESM11_ESM.pdf]

nature portfolio

Corresponding author(s): Olaf Blanke

Last updated by author(s): 2023.06.14

Reporting Summary

Nature Portfolio wishes to improve the reproducibility of the work that we publish. This form provides structure for consistency and transparency in reporting. For further information on Nature Portfolio policies, see our Editorial Policies and the Editorial Policy Checklist.

Statistics

For all statistical analyses, confirm that the following items are present in the figure legend, table legend, main text, or Methods section.

n/a	Confirmed	
☐	☒	The exact sample size (*n*) for each experimental group/condition, given as a discrete number and unit of measurement
☐	☒	A statement on whether measurements were taken from distinct samples or whether the same sample was measured repeatedly
☐	☒	The statistical test(s) used AND whether they are one- or two-sided<br>*Only common tests should be described solely by name; describe more complex techniques in the Methods section.*
☐	☒	A description of all covariates tested
☐	☒	A description of any assumptions or corrections, such as tests of normality and adjustment for multiple comparisons
☐	☒	A full description of the statistical parameters including central tendency (e.g. means) or other basic estimates (e.g. regression coefficient) AND variation (e.g. standard deviation) or associated estimates of uncertainty (e.g. confidence intervals)
☐	☒	For null hypothesis testing, the test statistic (e.g. *F*, *t*, *r*) with confidence intervals, effect sizes, degrees of freedom and *P* value noted<br>*Give P values as exact values whenever suitable.*
☒	☐	For Bayesian analysis, information on the choice of priors and Markov chain Monte Carlo settings
☒	☐	For hierarchical and complex designs, identification of the appropriate level for tests and full reporting of outcomes
☐	☒	Estimates of effect sizes (e.g. Cohen's *d*, Pearson's *r*), indicating how they were calculated

Our web collection on statistics for biologists contains articles on many of the points above.

Software and code

Policy information about availability of computer code

Data collection	Study 1: The experiment was implemented in Unity 3D 2019.3.13f1 (programming language: C#). The experiment was controlled and tracked through a smartphone companion application implemented in Unity 3D 2019.3.13f1 (programming language: C#). The robotic device application was implemented in a Visual studio 2019 solution (programming languages: C, C++). The executable build of the experiment and of the companion application have been deposited at https://doi.org/10.5281/zenodo.10511579 . The code of the robotic device application have been deposited at https://gitlab.epfl.ch/fbernasc/roboticsph .<br>Study 2: The online experiment was developed in house (programming languages and technologies used: on client side: javascript, html, css; on server side: https server in node.js, nginx as a reverse proxy, running in two docker containers). The stimuli used in study 2 have been deposited at https://doi.org/10.5281/zenodo.10511579 .
Data analysis	Data analysis scripts were written with R (4.2.2), and are available at https://doi.org/10.5281/zenodo.10511579 .

For manuscripts utilizing custom algorithms or software that are central to the research but not yet described in published literature, software must be made available to editors and reviewers. We strongly encourage code deposition in a community repository (e.g. GitHub). See the Nature Portfolio guidelines for submitting code & software for further information.

Data

Policy information about availability of data

All manuscripts must include a data availability statement. This statement should provide the following information, where applicable:

- Accession codes, unique identifiers, or web links for publicly available datasets
- A description of any restrictions on data availability
- For clinical datasets or third party data, please ensure that the statement adheres to our policy

The main data supporting the results in this study are available within the paper and its supplementary Information. The source data have been deposited at https://doi.org/10.5281/zenodo.10511579. Source data used to generate figures are provided with this paper as a Source Data file.

Research involving human participants, their data, or biological material

Policy information about studies with human participants or human data. See also policy information about sex, gender (identity/presentation), and sexual orientation and race, ethnicity and racism.

Reporting on sex and gender	Study 1: Twenty-height healthy participants (18 women, 10 men; age ranging from 18 to 33 years, mean ± SD age = 24 ± 3.42 years) took part in this experiment.<br>Study 2: One hundred and seventy patients with Parkinson's Disease (93 women, 77 men; age ranging from 42 to 79 years, mean ± SD age = 65.4 ± 7.83 years; Parkinson's Disease duration ranging from 1 month to 25.6 years, mean ± SD PD duration = 6.44 ± 5.19 years) took part in this study.
Reporting on race, ethnicity, or other socially relevant groupings	n.a.
Population characteristics	Study 1: Twenty-height healthy participants (18 women, 10 men; age ranging from 18 to 33 years, mean ± SD age = 24 ± 3.42 years) took part in this experiment. They were all right-handed according to the Edinburgh Handedness Inventory (score ranging from 50 to 100, mean ± SD score = 87.5 ± 18). None of the participants had current nor history of neurological, psychiatric and substance abuse disorders. Participants were also screened for a good stereoscopic vision threw a stereoscopic acuity test.<br>Study 2: One hundred and seventy patients with Parkinson's Disease (93 women, 77 men; age ranging from 42 to 79 years, mean ± SD age = 65.4 ± 7.83 years; Parkinson's Disease duration ranging from 1 month to 25.6 years, mean ± SD PD duration = 6.44 ± 5.19 years) took part in this study. At the beginning of the experiment, participants were asked to indicate their gender, age, country, time, visual disturbances, and whether they had been diagnosed with Parkinson disease. In case of positive answer to this last question, participants were also asked the date of diagnosis (year and month), the side of the body where symptoms appeared first ("left", "right", "both", "I don't know"), the current medication along with the daily dosage, and the time of the last medication intake for Parkinson's disease (Levodopa).
Recruitment	Study 1: Participants were recruited through an online recruiting system (https://lnco-geneva.sona-systems.com). Participants were rewarded for their time with monetary compensation (CHF20/hour).<br>Study 2: Participants were recruited with the help of Parkinson Schweiz (information and link on their website), Parkinson's UK (information and link on their website and in their newsletter) and Association France Parkinson (information and link on their website and in their newsletter).
Ethics oversight	Study 1: The experimental procedures (under protocol reference n° 2015-00092) were approved by the Cantonal Ethics Committee of Geneva (Commission Cantonale d'Ethique de la Recherche sur l'Être Humain - CCER). All the participants signed a written informed consent before participating in the experiment.<br>Study 2: This study was considered as falling outside of the scope of the swiss legislation regulating research on human subjects, so that the need for local ethics committee approval was waived (Commission Cantonale d'Ethique de la Recherche sur l'Être Humain - CCER, Switzerland – Req-2021-00378)). All participants consented to voluntarily participate in the study, prior to the beginning of the experiment.

Note that full information on the approval of the study protocol must also be provided in the manuscript.

Field-specific reporting

Please select the one below that is the best fit for your research. If you are not sure, read the appropriate sections before making your selection.

☐ Life sciences ☒ Behavioural & social sciences ☐ Ecological, evolutionary & environmental sciences

For a reference copy of the document with all sections, see nature.com/documents/nr-reporting-summary-flat.pdf

Behavioural & social sciences study design

All studies must disclose on these points even when the disclosure is negative.

Study description	In this manuscript, we establish a new digital quantitative marker for hallucinations by using a novel combination of virtual reality (VR), robotics, and digital online technology, based on data in healthy participants and in patients with Parkinson's Disease.
Research sample	Study 1: We tested healthy controls to develop and validate the novel protocol and implicit measure (human numerosity estimation task) to quantify robot-induced presence hallucination, without the possible confounds of verbal reports or confounds associated when presence hallucinations results from disease.<br>Study 2: We extended the numerosity estimation task to patients with Parkinson's Disease, who suffered from disease-related spontaneous presence hallucinations and investigated the clinical validity of the numerosity estimation measure.
Sampling strategy	Study 1: This study is preregistered at https://osf.io/yr3cp. A power analysis on the data obtained in a pilot experiment described in the preregistration suggested a sample size of 27 subjects (power 80%; alpha = 0.05). We round up to 28 participants to have an equal distribution of condition order across participants. The sample size estimation was conducted using R and the simr package. Participants were recruited through an online recruiting system (https://lnco-geneva.sona-systems.com). Participants registered to this online recruiting system could see the study and register to it on a voluntary basis. We stop the recruitment when 28 participants registered and participated in the study.<br>Study 2: The sample size was not predetermined, although we expected a large number of patients with Parkinson's Disease to participate in our online study. The recruitment was planned to be and thus was opened from August 2021 to June 2022. A total of 170 patients with Parkinson's disease took part in this study.
Data collection	Study 1: Participants were immersed in Virtual Reality, with minimized interactions with the real experimenter. The task instructions were provided in immersive Virtual Reality in a pre-recorded yet interactive manner, thereby automatizing the experimental procedure. Only the participant and the experimenter were present in the experimental room during the experiment.<br>Study 2: Parkinson's Disease patients performed the web-based digital task at home, by themselves on their own personal computer or tablet. They were instructed to perform the experiment while being alone in the room.
Timing	Study 1: This study was conducted from February 2021 to April 2021.<br>Study 2: This study was conducted from August 2021 to June 2022.
Data exclusions	Study 1: No data were excluded from the analysis.<br>Study 2: Of the 170 patients who participated in our online study, we included a total of 118 Parkinson's Disease patients in the analysis of the numerosity estimation task: 63 Parkinson's Disease patients with presence hallucinations (PD-PH) and 55 Parkinson's Disease patients without any hallucinations (PD-nH). The selection criteria are described below. Participants who had a very low refresh rate (less than 20Hertz) or resolution (less than 800px on both axis) were excluded from the analysis of the numerosity task. This resulted in the exclusion of respectively 4 and 4 participants. Participants who reported visual disturbances that could negatively impact the task were also excluded from the analysis of the numerosity task. This resulted in the exclusion of 10 participants. Participants who reported hallucinations, but not presence hallucination (passage hallucinations, visual illusions or structured visual hallucinations, n = 39) were also excluded from the current analysis. In the selection procedure described above, some participants belong to different categories of rejection criteria.
Non-participation	Study 1: No participant dropped out.<br>Study 2: 13 Parkinson's Disease patients (13/183 = 7%) stopped the experiment during the numerosity task. All drops were made during the first half of the numerosity estimation task. The reasons were: The experiment takes too much time (n=5) ; The experiment is boring (n=4) ; I am too tired to continue (n=1) ; Other (not indicated) (n=3).
Randomization	Participants were not allocated into experimental groups.

Reporting for specific materials, systems and methods

We require information from authors about some types of materials, experimental systems and methods used in many studies. Here, indicate whether each material, system or method listed is relevant to your study. If you are not sure if a list item applies to your research, read the appropriate section before selecting a response.

Materials & experimental systems

n/a	Involved in the study	
x		Antibodies
x		Eukaryotic cell lines
x		Palaeontology and archaeology
x		Animals and other organisms
x		Clinical data
x		Dual use research of concern
x		Plants

Methods

n/a	Involved in the study	
x		ChIP-seq
x		Flow cytometry
x		MRI-based neuroimaging
